# Distribution of serotypes and patterns of antimicrobial resistance among commensal *Streptococcus pneumoniae* in nine European countries

**DOI:** 10.1186/s12879-018-3341-0

**Published:** 2018-08-29

**Authors:** Rachid Y. Yahiaoui, Hester J. Bootsma, Casper D. J. den Heijer, Gerlinde N. Pluister, W. John Paget, Peter Spreeuwenberg, Krzysztof Trzcinski, Ellen E. Stobberingh

**Affiliations:** 1 Maastricht University Medical Centre/CAPHRI, Maastricht, The Netherlands; 2 Haga hospital, Department medical microbiology, The Hague, The Netherlands; 3 Centre for Infectious Diseases Research Control, National Institute for Public Health and the Environment, Bilthoven, The Netherlands; 4 NIVEL, The Netherlands Institute for Health Services Research, Utrecht, The Netherlands; 5 Department of Primary and Community Care, Radboud University Nijmegen Medical Centre, Nijmegen, The Netherlands; 6 Paediatric Immunology and Infectious Diseases, Wilhelmina Children’s Hospital, University Medical Centre Utrecht, Utrecht, The Netherlands

**Keywords:** *Streptococcus pneumoniae*, Vaccination, Carriage

## Abstract

**Background:**

*Streptococcus pneumoniae* is a commensal of the human upper respiratory tract and a major cause of morbidity and mortality worldwide. This paper presents the distribution of serotypes and antimicrobial resistance in commensal *S. pneumoniae* strains cultured from healthy carriers older than four years of age in nine European countries.

**Methods:**

Nasal swabs from healthy persons (age between 4 and 107 years old) were obtained by general practitioners from each country from November 2010 to August 2011. Swabs were cultured for *S. pneumoniae* using a standardized protocol. Antibiotic resistance was determined for isolated *S. pneumoniae* by broth microdilution. Capsular sequencing typing was used to identify serotypes, followed by serotype-specific PCR assays in case of ambiguous results.

**Results:**

Thirty-two thousand one hundred sixty-one nasal swabs were collected from which 937 *S. pneumoniae* were isolated. A large variation in serotype distribution and antimicrobial resistant serotypes across the participating countries was observed. Pneumococcal vaccination was associated with a higher risk of pneumococcal colonization and antimicrobial resistance independently of country and vaccine used, either conjugate vaccine or PPV 23).

**Conclusions:**

Serotype 11A was the most common in carriage followed by serotypes 23A and 19A. The serotypes showing the highest resistance to penicillin were 14 followed by 19A. Serotype 15A showed the highest proportion of multidrug resistance.

**Electronic supplementary material:**

The online version of this article (10.1186/s12879-018-3341-0) contains supplementary material, which is available to authorized users.

## Background

*Streptococcus pneumoniae* (pneumococcus) is a commensal of the human upper respiratory tract [1] and a major cause of morbidity and mortality worldwide. Pneumococcal disease has various manifestations including otitis media, pneumonia, septicemia and meningitis [2]. Incidence of the disease is highest at extremities of life: in very young children and in the elderly. Based on capsular polysaccharide chemistry and immunogenicity, over 90 distinct capsular types (serotypes) have been identified so far [3].

Two types of pneumococcal vaccines are commercially available: a pneumococcal polysaccharide vaccine (PPV) and pneumococcal conjugate vaccines (PCVs). Currently used PPV (Pneumovax 23) was first introduced in 1983 and by targeting 23 pneumococcal serotypes (1, 2, 3, 4, 5, 6B, 7F, 8, 9 N, 9 V, 10A, 11A, 12F, 14, 15B, 17F, 18C, 19A, 19F, 20, 22F, 23F, 33F) has valency broader than any PCV. However, unlike PCVs, PPV is not effective in very young children. Three different PCV’s are in use. The seven-valent vaccine (Prevnar, PCV7) was introduced in 2000, followed in 2009 by ten-valent (Synflorix, PCV10) and in 2010 by thirteen-valent (Prevenar13, PCV13) vaccines. PCV7 comprises serotypes 4, 6B, 9 V, 14, 18C, 19F and 23F, PCV10 includes additional serotypes 1, 5 and 7F, and PCV13 contains extra serotypes 3, 6A and 19A.

The introduction of PCVs into infant immunization programs has led to a major decrease in vaccine serotype (VT) disease in vaccinated children but to a various degree also resulted in herd effects across whole populations [4]. These indirect effects in unvaccinated individuals were caused by reduction of VT strain carriage in young children who are the main reservoir and main transmitters of pneumococci [5, 6]. PCVs may also contribute to a decrease in overall incidence of antimicrobial resistant pneumococcal disease [7]. This effect was augmented by the fact that VT strains were usually more resistant to antibiotics compared to non-vaccine (NVT) strains [7]. However, long term benefits of PCVs were eroded by the emergence of (multidrug-resistant) NVTs in carriage and in disease, the so called vaccine-induced serotype replacement [8].

There are differences in the timing of vaccine introduction, vaccination policies and vaccine coverage among countries that implemented PPV and PCVs into their National Immunization Programs (NIPs) [9]. This could result in differences in direct and herd effects, in serotype replacement and in resistance to anti-pneumococcal drugs in strains circulating in carriage and causing disease. By the end of 2012, 26 of the 53 countries of the European region vaccinated infants with PCVs in their NIPs [9]. In 2011, the PCVs coverage in infants’ vaccination (after administration of a minimum of 3 doses of vaccine by the age of 2 years) was more than 90% in these 26 European countries [9]. To our knowledge, there are no studies that evaluated the serotype distribution in commensal pneumococcal populations and its relationship with antimicrobial resistance across European countries as well as the impact of pneumococcal vaccination in a multicentre surveillance on pneumococcal carriage.

In this report, we assessed serotypes and resistance to antimicrobial agents of *S. pneumoniae* strains cultured from healthy carriers older than four years of age in nine European countries that differed in the timing of pneumococcal vaccines introduction, vaccines schedule and coverage, vaccines used (PCVs and PPV) and presence of catch-up campaigns. Results were analysed for impact of pneumococcal vaccination (immunization status) on individual person level and for effects that could be linked to differences in pneumococcal vaccination programs.

## Methods

### Study design

*S. pneumoniae* strains were cultured from samples collected from November 2010 to August 2011, as a part of the ‘The Appropriateness of prescribing antimicrobial agents in primary health care in Europe with respect to antimicrobial resistance’ (APRES) study as described by van Bijnen et al. [10]. Briefly, general practitioners (GP) from Austria, Belgium, Croatia, France Hungary, Spain, Sweden, the Netherlands and the United Kingdom (9 countries, 20 GPs per country), were each asked to provide nasal swabs from 200 healthy persons (with no history of antibiotic therapy or hospitalization in the previous three months), older than 4 years (except for UK, where for ethical reasons patients were older than 18 years). Within 48 h after collection swabs were transported to each national laboratory for further processing, with the exception of samples collected in France that were all sent to the Dutch national laboratory at Maastricht University Medical Centre (MUMC). On arrival to diagnostic labs, samples were cultured for *S. pneumoniae* using a standardized protocol [10]. Putative *S. pneumoniae* isolates from all participating countries were sent to the MUMC in skimmed milk at -80 °C for further analysis.

All participants provided written informed participatory consent and in the case of children aged less than 16, their parents or guardians provided written informed participatory consent on their behalf. All methods were approved by named institutional committee and were performed in accordance with relevant guidelines and regulations.

### Capsular sequence typing (CST)

CST was performed in the National Institute for Public Health and the Environment, Bilthoven, The Netherlands as previously described by Elberse et al. [11]. Briefly, culture of *S. pneumoniae* in Brain Heart Infusion broth with 0,5% yeast extract, incubated overnight at 37 °C and 5% CO_2_, was heated at 95 °C for 10 min and used as a DNA template in PCR to amplify a fragment of the capsular *wzh* gene. Amplicons were sequenced by BaseClear BV, Leiden, The Netherlands. Sequences generated were assembled, edited and trimmed using Bionumerics v6.1 (Applied Maths, Sint-Maartens-Latem, Belgium) and assigned a capsular sequence type (CT) using the CST database (https://www.rivm.nl/mpf/typingtool/spn/). CT is a composite assignment, in which the first part represents the serotype assessed by conventional serotyping (Quellung) followed by the number representing a consecutive *wzh* allele identified among strains of a given serotype [11]. When an allele not yet recorded in CST database was found, serotype of an isolate was determined by Quellung method at the Netherlands National Reference Laboratory for Bacterial Meningitis (NRBM), Amsterdam, the Netherlands.

For statistical analysis feasibility, isolates were grouped by serotype and not by individual CTs. For CTs associated with a single serotype (61 of the 79 CST types found in this study), grouping was based on the first part of the CST assignment. For CTs represented by multiple serotypes, particularly those concerning vaccine serotypes, additional PCR and/or PCR-sequencing assays were performed as follows. For CTs representing serogroup 6 isolates, distinction between 6A/B and 6C/D was made using primers specific for *wciNbeta,* after which 6A and 6B were distinguished by PCR sequencing of *wciP* as described [12]. For CTs 15B-01, 15C-01, 22F-01, 23F-01, and 24F-01, the appropriate serotype-specific primers from the protocol of the CDC for multiplex PCR serotype deduction (http://www.cdc.gov/streplab/pcr.html) were used in single PCR. For isolates of CT 34–01, PCR-sequencing of the *wzg* gene was performed to distinguish between serotype 17 and 34 isolates. Finally, distinction between CT 25F-02-associated serotypes 25A/F and 38 was achieved by PCR-amplification of the *wcyV* gene. When no gene was amplified with the used primers and no CST could not be assigned, isolates were considered as not typable. Primers used for these additional PCR assays are shown in Additional file 1: Table S5.

### Antimicrobial susceptibility

All isolates were tested for susceptibility to ceftazidime, clarithromycin, clindamycin, penicillin, tetracycline and trimethoprim-sulfamethoxazole. Minimal inhibitory concentrations of these drugs were assessed by broth microdilution method in accordance with EUCAST guidelines and EUCAST epidemiological cut-offs were applied as breakpoints [13]. Multidrug resistance was defined as resistance to three or more classes of antimicrobial agents.

### Data analysis

In order to study the effect of vaccination on pneumococcal carriage and antimicrobial resistance, a multilevel logistic regression was performed. To account for non-random clustering of our data at family level and to control whether age and gender affects pneumococcal carriage prevalence, a 3-level multilevel logistic regression model (country, GP and patient) was estimated using MLWIN software package. Statistical analysis was performed using the PASW software package 19.0, with *p*-value < 0.05 considered statistically significant.

## Results

### Participants and bacterial strains

Table 1 shows the demographic background of the participating individuals. In total, 31,625 individuals were recruited, varying from 3969 in Spain to 3025 in Belgium. The proportion of males ranged between 39.9% in Croatia and 45.6% in Belgium. Working in the healthcare sector ranged between 2.2% in Hungary to 16.5% in Sweden and working in nursery between 1% in Croatia to 4.1% in Sweden. Living with children < 5 years of age was between 10.1% in Belgium and 16.1% in Sweden. Among all participants, 937 were identified as pneumococcal carriers and ranged between170 in France to 36 in UK.

**Table 1:** Demographic overview of the participant individuals (in %)

	Austria	Belgium	Croatia	France	Hungary	Netherlands	UK	Spain	Sweden
Gender of patient									
Man	42.8	45.6	39.9	44.5	44.2	41.6	40.6	41.6	42.3
Women	55.8	53.9	58.5	54.5	54.2	55.7	58.8	58.4	57.2
Unknown	1.4	0.5	1.6	1.0	1.6	2.7	0.6	0.0	0.5
Age in year category									
4–9	1,2	1,3	6.0	3,3	8,5	3,2	0.0	4,9	5,4
10–17	2,2	3,1	8,2	4,7	15,6	5,3	0.0	5,9	5,4
18–29	15,8	8,2	9,2	11,5	9,6	11,1	14,7	9,2	9,6
30–60	50,2	40,9	38,8	45,2	36,5	45,7	46,3	41,5	37,7
> 60	29,8	46,5	37,4	34,9	29,1	33,9	38,8	38,5	41,6
Job in Healthcare sector									
Yes	6,0	4,6	3,7	3,1	2,2	10,3	8,8	3,3	16,5
Job in nursery									
Yes	1,9	2,2	1,0	2,1	3,4	3,0	2,4	2,0	4,1
None from the above-mentioned jobs or unknown									
Yes	92,1	93,2	95,3	94,8	94,4	86,7	88,8	94,8	79,4
Do you live with children of 5 years and younger?									
Yes	11,8	10,1	13,6	14,2	11,1	13,7	13,9	14,0	16,1
No	87,1	89,9	85,6	85,2	86,2	84,6	85,2	82,0	82,8
unknown	1,1	0,0	0,8	0,6	2,7	1,7	0,9	4,0	1,1

### Pneumococcal vaccination policy in the participating countries

Seven of the nine participating countries had introduced a pneumococcal conjugate vaccine in their NIP before November 2010 (Additional file 1: Table S1a) and used PCV7 during one (Sweden) to six years (Austria) prior to study onset. The exceptions were Croatia and Spain, two countries that only have risk-based pneumococcal vaccination programs. France was the only country with vaccination of risk group patients in the NIP in addition to infants. In Austria, Belgium, France and Sweden, PCV13 and PPV23 were used in adults and in risk populations (Additional file 1: Table S1b). In the Netherlands and Spain, only PPV23 was used in adult and on risk-based immunization. In Hungary, no recommendations were in place for vaccination of elderly and risk patients (Additional file 1: Table S1b).

### Vaccination status among participants

Among all participants, 10.3% (*n* = 3316) were vaccinated, 79.0% (*n* = 25,404) were not, and 10.7% (*n* = 3441) had an unknown vaccination status. Among carriers, Spain had the highest percentage of vaccinated individuals (30.5%) and Croatia the lowest (0.7%) (Table 2). In all countries except Austria and Belgium, participants between 4 and 9 years old were more frequently vaccinated than those older than 10 years (*p* <  0.0001; Additional file 1: Table S2).

**Table 2:** Vaccination status of participants per country

	Total participants population	Vaccinated participants N (%)	Non-vaccinated participants N (%)	Participants with unknown status N (%)	*S.pneumoniae* isolated N (%)	Vaccinated carriers %
Austria	3309	193 (5.8)	2254 (68.1)	862 (26.1)	38 (1.1)	5.3
Belgium	3025	493 (16.3)	2475 (81.8)	57 (1.9)	44 (1.5)	15.9
Croatia	3952	27 (0.7)	3763 (95.2)	162 (4.1)	134 (3.4)	0.7
France	3857	197 (5.1)	3368 (87.3)	292 (7.6)	170 (4.4)	13.5
Hungary	3832	134 (3.5)	3484 (90.9)	214 (5.6)	116 (3.0)	5.2
Netherlands	3847	89 (2.3)	3012 (78.3)	746 (19.4)	129 (3.4)	6.2
Spain	3969	1109 (27.9)	2691 (67.8)	169 (4.3)	167 (4.2)	30.5
Sweden	3214	304 (9.5)	2411 (75.0)	499 (15.5)	103 (3.2)	6.8
UK	3156	770 (24.4)	1946 (61.7)	440 (13.9)	36 (1.1)	27.8
Total	32,161	3316 (10.3)	25,404 (79.0)	3441 (10.7)	937 (2.9)	12.3

### Serotype carriage in the participating countries

Serotype 11A was the most common in carriage in the study population (*n* = 60) followed by serotypes 23A (*n* = 58), 19A (*n* = 52), 3 (*n* = 51), 6C (*n* = 44) and 23B (*n* = 39). All these serotypes were considered as non-PCV types since none was targeted by either PCV7 or PCV10 used in the study populations at the time of sample collection. These serotypes were followed by 19F (*n* = 38) and 23F (*n* = 37), targeted by all commercially available pneumococcal vaccines. Serotype 23F was the most frequent among *S. pneumoniae* carriage isolates in Croatia (*n* = 15 of 134, 11.2%) one of two countries without PCVs in NIP. Serotype 23F was along with serotype 11A (*n* = 11 of 103, 10.7% each) also most common in carriage in Sweden, country with shortest PCV immunization program at the time the study was conducted (Additional file 1: Table S1a and b). Serotype 6C was the most common in the Netherlands (n = 11 of 129, 8.5%) and Spain (n = 11 of 167, 6.6%). Serotype 3 (n = 6, 14.0%), 15A (n = 6, 12.8%), 11A (*n* = 18, 10.6%) and 10A (n = 5, 13.9%) were the most common in Austria, Belgium, France and UK, respectively. Serotypes 23A and 15 B/C (*n* = 9, 7.8%) were the most common in Hungary (Table 3 and Additional file 1: Table S3).

**Table 3:** Serotypes distribution by country (%). Serotypes listed in order from highest to lowest in frequency among all *S. pneumoniae* strains cultured in the study as reported in the last column

Serotype	Austria N = 38	Belgium N = 44	Croatia *N* = 134	France *N* = 170	Hungary *N* = 116	Netherlands *N* = 129	Spain *N* = 167	Sweden *N* = 103	UK *N* = 36	Total *N* = 937
11A	5.3	6.8	2.2	**10.6**	6.9	7.0	2.4	**10.7**	5.6	**6.4**
23A	2.6	2.3	6.7	7.6	**7.8**	4.7	4.8	7.8	8.3	6.2
19A	2.6	2.3	7.5	8.2	0.9	6.2	4.8	5.8	8.3	5.5
3	**15.8**	0.0	3.7	9.4	4.3	4.7	5.4	2.9	2.8	5.4
6C	5.3	4.5	3.0	5.3	0.9	**8.5**	**6.6**	1.9	5.6	4.7
NT	5.3	9.1	3.7	4.7	3.4	7.0	3.6	3.9	2.8	4.6
23B	5.3	6.8	0.0	7.1	0.0	7.0	5.4	1.9	5.6	4.2
19F	5.3	0.0	6.7	3.5	5.2	3.1	3.0	4.9	2.8	4.1
23F	5.3	0.0	**11.2**	0.0	0.9	2.3	1.2	**10.7**	8.3	3.9
15B/C	7.9	2.3	0.7	2.4	**7.8**	1.6	4.8	3.9	8.3	3.7
35B	2.6	2.3	2.2	5.3	6.9	1.6	3.6	1.9	5.6	3.6
35F	2.6	4.5	3.0	2.9	5.2	1.6	1.2	8.7	5.6	3.5
22F	2.6	4.5	1.5	4.7	2.6	3.9	2.4	4.9	5.6	3.4
6A	7.9	0.0	7.5	1.2	3.4	3.1	1.2	2.9	0.0	3.0
10A	0.0	2.3	0.0	1.2	6.0	4.7	4.2	0.0	**13.9**	3.0
6B	0.0	0.0	6.7	0.6	1.7	6.2	1.2	2.9	0.0	2.7
15A	2.6	**13.6**	0.7	3.5	0.0	1.6	3.6	1.9	0.0	2.6
17F	0.0	2.3	0.0	3.5	3.4	4.7	3.0	0.0	2.8	2.5
24F	0.0	4.5	0.0	2.9	5.2	0.8	4.2	0.0	0.0	2.2
18C	0.0	0.0	0.7	0.0	6.0	3.1	1.2	5.8	0.0	2.1
16F	0.0	2.3	0.7	0.6	4.3	0.8	5.4	1.0	0.0	2.0
7F	2.6	6.8	1.5	1.2	0.0	2.3	3.0	1.9	0.0	1.9
9 N	0.0	2.3	5.2	0.6	0.9	2.3	1.2	1.9	0.0	1.8
37	2.6	0.0	3.0	0.0	1.7	3.1	3.0	1.0	0.0	1.8
14	0.0	2.3	3.7	0.0	1.7	0.0	4.2	1.0	0.0	1.7
33F	0.0	0.0	0.0	2.4	0.9	2.3	1.8	3.9	0.0	1.6
34	0.0	0.0	2.2	0.0	3.4	0.8	1.8	1.9	0.0	1.4
21	0.0	0.0	0.0	3.5	0.9	0.0	3.0	0.0	2.8	1.4
31	0.0	9.1	0.7	1.2	2.6	0.0	1.2	0.0	0.0	1.3

*UK* United Kingdom; in bold: serotypes with the highest frequency in a country, *NT* not typable. Only serotypes represented by more than 10 strains among all *S. pneumoniae* strains cultured in the study are reported. Complete overview of serotypes is shown in Additional file 1: Table S3

Serotypes 17F and 22F, both targeted by PPV23 vaccine, were significantly associated with age older than 10 years (*p* = 0.03 and *p* = 0.01, respectively). Serotype 23F (PCV7 serotype) was significantly associated with age 4–9 years (*p* = 0.01). Other serotypes were not associated with any age category (Additional file 1: Table S4).

### Vaccination effect on pneumococcal colonization and serotypes

Among the pneumococcal carriers (all serotypes considered), 115 were vaccinated and 727 were not vaccinated, while the vaccination status was unknown for 95 individuals.

Table 4 shows the effect of pneumococcal vaccination on pneumococcal carriage. Being vaccinated was associated with a higher risk of pneumococcal colonization*.* None of the variables i.e. vaccine regimen, the presence of a catch-up campaign, the year of the vaccine implementation, the vaccine type, or the extent of vaccination program had separately a significant effect on pneumococcal colonization. Of 937 pneumococcal strains cultured from carriers, 170 (18.1%) were of PCV10 types. Of these 149 (15.9% of the total number of strains) were of PCV7. There were significantly fewer carriers of PCV10 serotypes among vaccinated (11 of 115, 9,6%) compared to non-vaccinated individuals (138 of 727, 19.0%), *p* = 0.01 (Table 5). These findings in combination with Table 4 suggest that vaccinated individuals have more chance to be colonized by non-vaccine serotypes.

**Table 4:** Relationship between vaccine-related variables (number of doses, presence of catch up campaign, year of implementation, vaccine type and extent of vaccination program), and pneumococcal carriage in the participating individuals

Models	Reference variable	Comparative variables	OR*	*p* value**
Basic model:				
Model 1: vaccination	Vaccinated	Not vaccinated	0.70 (0.55–0.89)	0.00**
		Unknown vaccination status	0.76 (0.54–1.05)	0.10
Models after addition vaccination program characteristics separately:				
Model 2: vaccine dose	2 + 1^b^	3 + 1^a^	1.39 (0.66–2.92)	0.37
Model 3: catch up campaign conduction	Catch up campaign conducted^c^	No catch up campaign conducted^d^	0.76 (0.54–1.05)	0.41
Model 4: period since the vaccine implementation	implemented for one year	implemented for more than one year	1.05 (0.88–1.25)	0.53
Model 5: type of vaccine	PCV 7	PCV 7, PCV10 and PCV 13	1.00 (0.39–2.55)	0.98
		PCV 10 and PCV 13	0.80 (0.31–1.87)	0.65
Model 6: extent of vaccination program	risk based and universal ^e^	Risk based ^g^	0.57 (0.17–1.87)	0.36
		Universal ^f^	0.43 (0.14–1.33)	0.14

*OR adjusted for countries, age and general practitioner; **p <  0.05 is significant

**Table 5:** distribution of vaccine and non-vaccine serotypes in vaccinated and non-vaccinated among the study population

Serotype carried	Vaccinated	Non-vaccinated	Unknown	Total
PCV7 serotypes ^a^	9	121	19	149
PCV10-PCV7 serotypes ^b^	2	17	2	21
PCV13-PCV10 serotypes ^c^	15	103	13	131
No vaccine type or PCV serotypes ^d^	0	2	0	2
Non-PCV13 vaccine types	88	477	61	626
PPV23 serotypes ^f^	50	396	54	500
Non-PPV23 vaccine types	65	331	41	437
Not determinate	1	7	0	8

^a^serotypes 4, 6B, 9V, 14, 18C, 19F and 23F; ^b^ serotypes 1, 5 and 7F; ^c^ serotypes 3, 6A and 19A; ^d^ serotypes 18For 18C (the serotyping method is not able to differentiate between serotypes 18F(NVT) and 18C (VT)); ^f^ serotypes 1, 2, 3, 4, 5, 6B, 7F, 8, 9N, 9V, 10A, 11A, 12F, 14, 15B, 17F, 18C, 19A, 19F, 20, 22F, 23F, 33F

Table 6 shows the antimicrobial resistance per serotypes*.* The highest resistance proportion to ceftazidime and penicillin was noticed among serotype 14 strains (13 of 16, 81.3%). Serotype 14 was the most frequent serotype showing resistance to penicillin, followed by serotype 19A and 15A. Among serotypes with more than 10 isolates, paediatric serotypes (6B, 9 V, 14, 19F and 23F) were more resistant to antimicrobial agents than non-paediatric serotypes (1, 3, 4, 7F). The serotypes presenting the highest proportion of multidrug resistance were 15A followed by 19A and 14. Serotypes 6C, 23B, 15A, 19A, 6A and 19F were significantly more prevalent whereas serotypes 22F, 23A, 3 and 14 were significantly less prevalent in the multidrug resistant fraction compared to the total study collection (Table 6).

**Table 6:** Distribution of antimicrobial resistance by serotype. Only serotypes represented by more than 10 isolates are reported. Correlation between frequency of serotypes in study population and in MDR fraction is given in the last column

serotypes	N (%)	Ceftazidime (%)	Clarithromycin (%)	Clindamycin (%)	Penicillin (%)	Tetracyclin (%)	Trimethropim/ sulfamethoxazole (%)	MDR Fraction N (%)	*p* value
11A	60 (6.4)	8.3	6.7	3.3	5.0	0.0	10.0	5 (8,3)	0.090
23A	58 (6.2)	0.0	19.0	19.0	1.7	19	24.1	2 (3,4)	0.007*
19A	52 (5.5)	67.3	53.8	50	67.3	**55.8**	**30.8**	32 (61,5)	< 0.001*
3	50 (5.3)	0.0	0.0	0.0	0.0	0.0	2.0	0 (0,0)	0.001*
6C/D	44 (4.7)	6.8	29.5	27.3	27.3	25.0	0.0	11 (25,0)	0.001*
23B	39 (4.2)	12.8	0.0	0.0	30.8	0.0	28.2	11 (28,2)	0.093
19F	37 (3.9)	32.4	56.8	48.6	37.8	54.1	18.9	14 (37,8)	0.001*
23F	37 (3.9)	13.5	16.2	10.8	16.2	10.8	16.2	6 (16,2)	0.934
15B/C	35 (3.7)	2.9	8.6	0.0	22.9	11.4	31.4	3 (8,6)	0.255
35B	34 (3.6)	38.2	8.8	5.9	38.2	2.9	5.9	3 (8,8)	0.283
35F	33 (3.5)	6.1	3.0	3.0	0.0	0.0	0.0	1 (3,0)	0.050
22F	32 (3.4)	0.0	0.0	0.0	0.0	0.0	0.0	0 (0,0)	0.017*
6A	28 (3.0)	39.3	39.3	10.7	39.3	10.7	25.0	10 (35,7)	0.015*
10A	28 (3.0)	7.1	10.7	7.1	7.1	10.7	10.7	2 (7,1)	0.243
6B	25 (2.7)	24.0	32.0	28.0	20.0	28.0	24.0	8 (32,0)	0.081
15A	24 (2.6)	58.3	**62.5**	**62.5**	62.5	50	8.3	15 *(62,5)*	< 0.001*
17F	23 (2.5)	8.7	4.3	4.3	8.7	4.3	0.0	1 (4,3)	0.172
24F	21 (2.2)	28.6	28.6	28.6	28.6	28.6	19.0	6 (28,6)	0.260
18C	20 (2.1)	0.0	0.0	0.0	0.0	0.0	10.0	0 (0,0)	0.079
16F	19 (2.0)	0.0	15.8	15.8	0.0	15.8	42.1	1 (5,3)	0.282
37	17 (1.8)	0.0	0.0	0.0	0.0	0.0	5.9	0 (0,0)	0.117
7F	17 (1.8)	0.0	0.0	0.0	0.0	0.0	0.0	0 (0,0)	0.117
9 N	17 (1.8)	0.0	0.0	0.0	0.0	0.0	0.0	0 (0,0)	0.117
14	16 (1.7)	**81.3**	31.3	25.0	**81.3**	18.8	43.8	8 (50,0)	0.001*
33F	15 (1.6)	0.0	33.3	33.3	0.0	26.7	6.7	1 (6,7)	0.462
21	13 (1.4)	0.0	0.0	0.0	7.7	0.0	0.0	0 (0,0)	0.201
31	13 (1.4)	0.0	0.0	0.0	0.0	0.0	0.0	0 (0,0)	0.201
34	13 (1.4)	61.5	0.0	0.0	30.8	0.0	23.1	0 (0,0)	0.201

*p < 0.05 is significant; MDR = Multidrug resistance; In bold: serotypes presenting the most MDR isolates; underlined: highly resistance

### Vaccination effect on antimicrobial resistance carriage

Table 7 shows the effect of vaccination on carriage of antimicrobial resistant pneumococcal isolates. Being vaccinated enhanced carriage of isolates resistant to at least one of the tested antimicrobial agents (model 1, OR = 0.60, *p* = 0.03). After adding different vaccination program characteristics (vaccine dose, conduction of catch-up campaign, period since vaccine was implemented, vaccine type and extent of vaccination program) separately, none of these could explain alone the vaccination effect (models 2–6).

**Table 7:** Effect of pneumococcal vaccination and vaccination variables (dose, conduction of catch up campaign, year of implementation, vaccine type and extent of vaccination program), on carriage of antimicrobial resistant pneumococcus (resistant to at least one antibiotic) in the participating individuals

Models	Reference variable	Comparative variables	OR*	*p* value**
Basic model:				
Model 1: vaccination	Vaccinated	Not vaccinated	0.60 (0.37–0.96)	0.03**
		Unknown vaccination status	0.63 (0.33–1.18)	0.15
Models after addition vaccination program characteristics separately:				
Model 2: vaccine dose	2 + 1^b^	3+1^a^	0.91 (0.35–2.32)	0.84
Model 3: catch up campaign conduction	Catch up campaign conducted ^c^	No catch up campaign conducted ^d^	1.57 (0.65–3.79)	0.32
Model 4: period since vaccine implementation	implemented for one year	implemented for more than one year	1.10 (0.90–1.34)	0.34
Model 5: vaccine type	PCV 10	PCV 7	1.23 (0.39–3.87)	0.72
		PCV 13	0.87 (0.27–2.72)	0.81
Model 6: extent of vaccination program	risk based and universal ^e^	Universal ^f^	0.69 (0.17–2.81)	0.61
		Risk based ^g^	1.07 (0.24–4.69)	0.92

*p < 0.05 is significant

^a^) Austria, Hungary, Netherlands, Spain, ^b^) Belgium, France, Hungary, Sweden, UK, ^c^) Belgium, FR, Hungary, UK, ^d^) Austria, Hungary, Netherlands, Spain, Sweden, ^e^) France, ^f^) Austria, Belgium, Hungary, Netherlands, Sweden, UK, ^g^) Hungary, Spain

## Discussion

In this report, we assessed serotypes and resistance to antimicrobial agents of *S. pneumoniae* strains cultured from healthy carriers older than four years of age in nine European countries that differed in the timing of pneumococcal vaccines introduction. A large variation was found in serotype distribution in the participating countries and we observed difference in antimicrobial resistance including multidrug resistance, among these serotypes. The major finding was that pneumococcal vaccination was associated with a high risk of non PCV10 serotypes carriage.

This study was carried out on a large and well documented population, covering different age groups. To eliminate intra-laboratory variations, all methods were conducted in a central laboratory per method (susceptibility testing in MUMC, molecular serotyping in RIVM and conventional serotyping using Quellung method in NRBM). These points allowed us to accurately address the goals of this study. However, drawbacks were differences in numbers of strains collected and tested per country with significantly fewer strains from UK, Austria and Belgium (*p* <  0.0001) compared to any other participating sites. This could limit the generalization of our findings to the entire population in those countries. Possible explanation for differences in prevalence of carriage could be differences in age of sampled individuals (e.g. no minors under age of 18 years were sampled in UK) or in patterns of social contacts with very young children: carriage rates in parents of young children are reported to be few-fold higher compared to childless adults [14].

This study cannot be used to assess the spread and antimicrobial resistance in *S. pneumoniae* among non-vaccinated persons. This would require a longitudinal study. Furthermore, many factors determine carriage and resistance (e.g. antibiotic use was exclusion criterium in this study, seasonal variations in pneumococcal carriage), so one needs to be careful with drawing general conclusion based on these results.

For feasibility and costs considerations, capsular sequence typing was used in the primary method of serotyping. When highly frequent or vaccine CST’s could not be differentiated, additional PCR’s were performed. Conventional serotyping with type-specific sera was performed only when results generated with the CST method were equivocal. Our results support CST as a method alternative to the conventional Quellung serotyping in epidemiological studies.

PCV-7 was licensed in Europe in 2001. Higher valency vaccines were introduced since then (PCV-10 and PCV-13 in 2009 and 2010, respectively). If our results represented the effects of any vaccine in the studied population, these should be likely indirect (herd) effects of PCV7, and without any contribution of PCV13 since this vaccine was implemented after our study onset.

The most frequent serotypes among the carriage isolates were 11A, 19A, 3, 6C, 23A and 23B. All these serotypes were PPV vaccine or non-vaccine types. The most prevalent PCV7 vaccine type was 19F. These results might be explained by a replacement in the serotypes carriage. Our results are in concordance with an earlier report have reporting the predominance of non-vaccine serotypes, among which serotype 11A, in 336 paediatric patients in Ireland [15].

In contrast to previous carriage studies [16–18] which reported a decrease in pneumococcal carriage after vaccine implementation, our analyses show a higher risk of pneumococcal colonization upon vaccination. Most of the studies dealing with the effect of vaccination on pneumococcal carriage were conducted in vaccinated children and within few years after vaccination [19–24] . This reflects probably the immediate immunity and indirect effect of vaccination on the population. Our findings might be explained by the emergence of non-vaccine serotypes that co-circulated but were suppressed by vaccine serotypes. Another possible explanation could be that the protective effect of vaccination against pneumococcal carriage might decrease over time, allowing a re-emergence of vaccine serotypes in vaccinated patients. These results are supported by Principi et al., [25] who found that pneumococcal prevalence was higher in vaccinated individuals than in unvaccinated ones in a study enrolling 2076 children and adolescents from Italy.

Our results have shown that pneumococcal vaccination was associated with an increase in the prevalence of pneumococcal antimicrobial resistance. This may be explained by the fact that a vaccination might facilitate the introduction of new pneumococcal serotypes which are more resistant to antimicrobial agents [26] due to the replacement of vaccine serotypes [27].

A large variation in serotype distribution as well as in antimicrobial resistant serotypes was observed in the participating countries. In some countries, some serotypes were associated to resistance (19A and 14 resistance penicillin and ceftazidime). This might be due to differences in antimicrobial agents use between the participating countries [28], clonal spread of resistant microorganisms and antimicrobial cross-resistance between members of antimicrobial agents classes. This variation might justify the necessity of implementation of guidelines on antimicrobial agents use at country level.

Serotype 19A, a PCV-13 vaccine serotype, is the most frequent causative agent of invasive pneumococcal diseases All participants were provided a written consent form. For children consent was obtained from one of the parents or guardians. [29]. In our study, this serotype was also one of the most frequent carriage isolates (5.5%, *n* = 52) and one of the most resistant serotype to all antimicrobial agents tested. These results are in accordance with earlier reports [30].

Hackel et al., reported that serotype 15A was highly resistant to erythromycin and penicillin in clinical strains worldwide [31]. In our study, 15A representing 2.6% of all pneumococcal isolates (*n* = 24), was one of the most resistant serotypes to penicillin, ceftazidime, clarithromycin, clindamycin and tetracycline. Serotype 35B was, after 15A, the second most resistant non-vaccine serotype to penicillin and ceftazidime (38.2% of serotype 35B strains were resistant for both antibiotics). In the US, this serotype is becoming the dominating serotype in carriage and invasive pneumococcal disease, This is due to a clonal shift after the implementation of PCV7 and a spread of a β –lactam resistant clonal complex after the implementation of PCV13 [8, 32].

## Conclusions

In conclusion, pneumococcal vaccination is associated with a higher risk of non PCV10 serotypes colonization and antimicrobial resistance independently of country and vaccine used. Serotypes 14 (PCV-7), 15A (non-vaccine serotype) and 19A (PCV-13) had the highest proportion of antimicrobial resistance and multidrug resistance. The emergence of new serotypes and related prevalence of antimicrobial resistance might justify at the short-term, a continuous evaluation and adjustment of available vaccines, in order to include newly emerged serotypes. At the long-term, the implementation of new vaccines that could cover all pneumococcal serotypes such as whole cell vaccines, might be helpful.

## Additional file

Additional file 1: **Table S1.** a: Infants vaccination in National Immunization Program in the nine participating countries. b: Vaccination in elderly and risk patients in the nine participating countries**. Table S2.** Association between age and vaccination status in (non-) vaccinated participants in participants with known age range. **Table S3.** Serotypes distribution by country (%). Serotypes listed in order from highest to lowest in frequency among all *S. pneumoniae* strains cultured in the study as reported in the last column. **Table S4.** Serotypes distribution by age category (%). Serotypes listed in order from highest to lowest in frequency. Only serotypes represented by more than 10 strains among all *S. pneumoniae* strains cultured in the study are reported. Correlation between serotypes frequency in different age groups is shown in the last column. **Table S5.** Primers used in this study. (DOCX 67 kb)

## Abbreviations

APRES: “The Appropriateness of prescribing antimicrobial agents in primary health care in Europe with respect to antimicrobial resistance” study
CDC: Centers for Disease Control and Prevention
CST: Capsular Sequence Typing
CT: capsular sequence type
EUCAST: European Committee on Antimicrobial Susceptibility Testing
GP: general practitioners
NIP: National Immunization Programs
NVT: strains non-vaccine type
PCR: Polymerase Chain Reaction
PCV: pneumococcal conjugate vaccine
PPV: pneumococcal polysaccharide vaccine
RIVM: The National Institute for Public Health and the Environment
VT: vaccine serotype
