# Supplementary material for: Distribution of serotypes and patterns of antimicrobial resistance among commensal Streptococcus pneumoniae in nine European countries
Source: BMC Infect Dis. 2018 Aug 29;18:440. doi: 10.1186/s12879-018-3341-0 (PMC6116386; doi:10.1186/s12879-018-3341-0)
Supplement: Supplementary file 1 — Table S1. a: Infants vaccination in National Immunization Program in the nine participating countries. b: Vaccination in elderly and risk patients in the nine participating countries. Table S2. Association between age and vaccination status in (non-) vaccinated participants in participants with known age range. Table S3. Serotypes distribution by country (%). Serotypes listed in order from highest to lowest in frequency among all S. pneumoniae strains cultured in the study as reported in the last column. Table S4. Serotypes distribution by age category (%). Serotypes listed in order from highest to lowest in frequency. Only serotypes represented by more than 10 strains among all S. pneumoniae strains cultured in the study are reported. Correlation between serotypes frequency in different age groups is shown in the last column. Table S5. Primers used in this study. (DOCX 67 kb) [file 12879_2018_3341_MOESM1_ESM.docx]

**Supplementary tables:**

Distribution of serotypes and patterns of antimicrobial resistance among commensal *Streptococcus pneumoniae* in nine European countries

Rachid Y. Yahiaoui^1,5*^, Hester J. Bootsma^4^, Casper D.J. den Heijer^1^, Gerlinde N. Pluister^4^, W. John Paget^2,3^, Peter Spreeuwenberg^2^, Krzysztof Trzcinski^4,6^, Ellen E. Stobberingh^1,4^

^1^ Maastricht University Medical Centre/CAPHRI, Maastricht, The Netherlands

^2^ NIVEL, The Netherlands Institute for Health Services Research, Utrecht, The Netherlands

^3^ Department of Primary and Community Care, Radboud University Nijmegen Medical Centre, Nijmegen, The Netherlands

^4^ Centre for Infectious Diseases Research Control, National Institute for Public Health and the Environment, Bilthoven, The Netherlands

^5^ Haga hospital, Dept. medical microbiology, The Hague, The Netherlands

^6^ Paediatric Immunology and Infectious Diseases, Wilhelmina Children’s Hospital, University Medical Centre Utrecht, Utrecht, The Netherlands

*Corresponding author

Phone: +31702102260

e-mail: r.yahiaoui@hagaziekenhuis.nl

**Table S1a:** Infants vaccination in National Immunization Program in the nine participating countries [1-6]

| **Country** | **Vaccine type** | **Schedule** | **Extent of vaccination program** | **Catch up campaign** | **Year of implementation** | | |
| --- | --- | --- | --- | --- | --- | --- | --- |
|  |  |  |  |  | **PCV7** | **PCV10** | **PCV13** |
| Austria | PCV7, PCV10 PCV13 | 3+1 | Universal | No | 2004 | 2010 | 2009 |
| Belgium | PCV7, PCV13 | 2+1 | Universal | Yes (2007) | 2005 |  | 2011 |
| Croatia | PCV7 | 3+1 | Risk based | No | 2006 | - | - |
| France | PCV7, PCV13 | 2+1 | Universal + risk-based | Yes (2010) | 2006 | - | 2010 |
| Hungary | PCV7, PCV13 | 2+1 | Universal | Yes (2008) | 2008 | - | 2010 |
| Netherlands | PCV7, PCV10 | 3+1 | Universal | No | 2006 | 2011 |  |
| Spain | PCV7, PCV10, PCV13 | 3+1 | Risk based | No | 2001 | 2009 | 2010 |
| Sweden | PCV7, PCV10 PCV13 | 2+1 | Universal | No | 2009 | 2009 | 2010 |
| UK | PCV7 PCV13 | 2+1 | Universal | Yes (2006) | 2006 |  | 2010 |

**Table S1b:** Vaccination in elderly and risk patients in the nine participating countries [1-6].

| **Country** | **Vaccine type** | **Date of implementation of recommendation** | **Age based** | **Date of implementation PPV23** |
| --- | --- | --- | --- | --- |
| Austria | PCV13, PPV23 | 2014 | ≥50 years | 2003 |
| Belgium | PCV13, PPV23 | 2013 | ≥65 years | 1993 |
| Croatia | - | - | - | - |
| France | PCV13, PPV23 | 2013 | No | - |
| Hungary | - | - | - | - |
| Netherlands | PPV23 | - | - | - |
| Spain | PPV23 | 2001 | - | 1999 |
| Sweden | PPV23 | 1994 | ≥65 years | 1994 |
| UK | PCV13, PPV23 | 1992 | ≥65 years | 1992 |

**Table S2:** Association between age and vaccination status in (non-) vaccinated participants in participants with known age range

|  | 4-9 years | | Older than 10 years | | Unknown age | | p-value |
| --- | --- | --- | --- | --- | --- | --- | --- |
|  | Vaccinated | Non-vaccinated | Vaccinated | Non-vaccinated | Vaccinated | Non-vaccinated |  |
| Austria | 3 | 31 | 189 | 2203 | 1 | 20 | 0.9028 |
| Belgium | 4 | 32 | 488 | 2442 | 1 | 1 | 0.507 |
| Croatia | 7 | 209 | 20 | 3542 | 0 | 12 | < 0.0001* |
| France | 57 | 65 | 139 | 3291 | 1 | 12 | < 0.0001* |
| Spain | 89 | 103 | 1020 | 2588 | 0 | 23 | < 0.0001* |
| Netherlands | 14 | 83 | 75 | 2909 | 0 | 20 | < 0.0001* |
| Hungary | 24 | 289 | 110 | 3172 | 0 | 0 | 0.0002* |
| Sweden | 8 | 147 | 294 | 2259 | 2 | 5 | 0.0209* |
| UK | 0 | 0 | 768 | 1945 | 2 | 1 | nd |
| Total | 206 | 959 | 3103 | 24351 | 7 | 94 | p<0.0001* |

*P<0.05 is significant; nd=not determined

**Table S3:** Serotypes distribution by country (%). Serotypes listed in order from highest to lowest in frequency among all *S. pneumoniae* strains cultured in the study as reported in the last column.

| Serotype | Austria  N=38 | Belgium  N=44 | Croatia  N=134 | France  N=170 | Hungary  N=116 | Netherlands  N=129 | Spain  N=167 | Sweden  N=103 | UK  N=36 | Total  N=937 |
| --- | --- | --- | --- | --- | --- | --- | --- | --- | --- | --- |
| 11A | 5.3 | 6.8 | 2.2 | **10.6** | 6.9 | 7.0 | 2.4 | **10.7** | 5.6 | **6.4** |
| 23A | 2.6 | 2.3 | 6.7 | 7.6 | **7.8** | 4.7 | 4.8 | 7.8 | 8.3 | 6.2 |
| 19A | 2.6 | 2.3 | 7.5 | 8.2 | 0.9 | 6.2 | 4.8 | 5.8 | 8.3 | 5.5 |
| 3 | **15.8** | 0.0 | 3.7 | 9.4 | 4.3 | 4.7 | 5.4 | 2.9 | 2.8 | 5.4 |
| 6C | 5.3 | 4.5 | 3.0 | 5.3 | 0.9 | **8.5** | **6.6** | 1.9 | 5.6 | 4.7 |
| NT | 5.3 | 9.1 | 3.7 | 4.7 | 3.4 | 7.0 | 3.6 | 3.9 | 2.8 | 4.6 |
| 23B | 5.3 | 6.8 | 0.0 | 7.1 | 0.0 | 7.0 | 5.4 | 1.9 | 5.6 | 4.2 |
| 19F | 5.3 | 0.0 | 6.7 | 3.5 | 5.2 | 3.1 | 3.0 | 4.9 | 2.8 | 4.1 |
| 23F | 5.3 | 0.0 | **11.2** | 0.0 | 0.9 | 2.3 | 1.2 | **10.7** | 8.3 | 3.9 |
| 15B/C | 7.9 | 2.3 | 0.7 | 2.4 | **7.8** | 1.6 | 4.8 | 3.9 | 8.3 | 3.7 |
| 35B | 2.6 | 2.3 | 2.2 | 5.3 | 6.9 | 1.6 | 3.6 | 1.9 | 5.6 | 3.6 |
| 35F | 2.6 | 4.5 | 3.0 | 2.9 | 5.2 | 1.6 | 1.2 | 8.7 | 5.6 | 3.5 |
| 22F | 2.6 | 4.5 | 1.5 | 4.7 | 2.6 | 3.9 | 2.4 | 4.9 | 5.6 | 3.4 |
| 6A | 7.9 | 0.0 | 7.5 | 1.2 | 3.4 | 3.1 | 1.2 | 2.9 | 0.0 | 3.0 |
| 10A | 0.0 | 2.3 | 0.0 | 1.2 | 6.0 | 4.7 | 4.2 | 0.0 | **13.9** | 3.0 |
| 6B | 0.0 | 0.0 | 6.7 | 0.6 | 1.7 | 6.2 | 1.2 | 2.9 | 0.0 | 2.7 |
| 15A | 2.6 | **13.6** | 0.7 | 3.5 | 0.0 | 1.6 | 3.6 | 1.9 | 0.0 | 2.6 |
| 17F | 0.0 | 2.3 | 0.0 | 3.5 | 3.4 | 4.7 | 3.0 | 0.0 | 2.8 | 2.5 |
| 24F | 0.0 | 4.5 | 0.0 | 2.9 | 5.2 | 0.8 | 4.2 | 0.0 | 0.0 | 2.2 |
| 18C | 0.0 | 0.0 | 0.7 | 0.0 | 6.0 | 3.1 | 1.2 | 5.8 | 0.0 | 2.1 |
| 16F | 0.0 | 2.3 | 0.7 | 0.6 | 4.3 | 0.8 | 5.4 | 1.0 | 0.0 | 2.0 |
| 7F | 2.6 | 6.8 | 1.5 | 1.2 | 0.0 | 2.3 | 3.0 | 1.9 | 0.0 | 1.9 |
| 9N | 0.0 | 2.3 | 5.2 | 0.6 | 0.9 | 2.3 | 1.2 | 1.9 | 0.0 | 1.8 |
| 37 | 2.6 | 0.0 | 3.0 | 0.0 | 1.7 | 3.1 | 3.0 | 1.0 | 0.0 | 1.8 |
| 14 | 0.0 | 2.3 | 3.7 | 0.0 | 1.7 | 0.0 | 4.2 | 1.0 | 0.0 | 1.7 |
| 33F | 0.0 | 0.0 | 0.0 | 2.4 | 0.9 | 2.3 | 1.8 | 3.9 | 0.0 | 1.6 |
| 34 | 0.0 | 0.0 | 2.2 | 0.0 | 3.4 | 0.8 | 1.8 | 1.9 | 0.0 | 1.4 |
| 21 | 0.0 | 0.0 | 0.0 | 3.5 | 0.9 | 0.0 | 3.0 | 0.0 | 2.8 | 1.4 |
| 31 | 0.0 | 9.1 | 0.7 | 1.2 | 2.6 | 0.0 | 1.2 | 0.0 | 0.0 | 1.3 |
| 8 | 0.0 | 2.3 | 2.2 | 0.0 | 1.7 | 1.6 | 0.0 | 0.0 | 2.8 | 1.0 |
| 38 | 2.6 | 2.3 | 0.7 | 1.8 | 0.0 | 0.8 | 0.6 | 0.0 | 0.0 | 0.9 |
| 12F | 2.6 | 2.3 | 0.0 | 1.2 | 0.9 | 0.0 | 0.6 | 0.0 | 0.0 | 0.6 |
| 28A | 2.6 | 0.0 | 2.2 | 0.0 | 0.0 | 0.8 | 0.0 | 0.0 | 0.0 | 0.5 |
| 4 | 0.0 | 0.0 | 2.2 | 0.6 | 0.0 | 0.8 | 0.0 | 0.0 | 0.0 | 0.5 |
| 9V | 0.0 | 0.0 | 0.7 | 0.6 | 0.0 | 0.0 | 0.6 | 1.9 | 0.0 | 0.5 |
| 13 | 0.0 | 0.0 | 0.7 | 0.0 | 0.9 | 0.0 | 1.8 | 0.0 | 0.0 | 0.5 |
| 1 | 0.0 | 0.0 | 0.7 | 0.0 | 0.0 | 0.0 | 1.2 | 0.0 | 0.0 | 0.3 |
| 20 | 0.0 | 0.0 | 0.0 | 1.2 | 0.0 | 0.0 | 0.6 | 0.0 | 0.0 | 0.3 |
| 42 | 0.0 | 0.0 | 1.5 | 0.0 | 0.0 | 0.0 | 0.0 | 0.0 | 0.0 | 0.2 |
| 18F or 18C | 0.0 | 0.0 | 0.7 | 0.6 | 0.0 | 0.0 | 0.0 | 0.0 | 0.0 | 0.2 |
| 7C | 0.0 | 0.0 | 0.0 | 0.0 | 0.0 | 0.0 | 0.6 | 0.0 | 2.8 | 0.2 |
| 10B | 0.0 | 0.0 | 0.0 | 0.0 | 0.0 | 0.0 | 0.0 | 1.0 | 0.0 | 0.1 |
| 29 | 2.6 | 0.0 | 0.0 | 0.0 | 0.0 | 0.0 | 0.0 | 0.0 | 0.0 | 0.1 |
| 17A | 2.6 | 0.0 | 0.0 | 0.0 | 0.0 | 0.0 | 0.0 | 0.0 | 0.0 | 0.1 |
| 15F | 2.6 | 0.0 | 0.0 | 0.0 | 0.0 | 0.0 | 0.0 | 0.0 | 0.0 | 0.1 |
| 27 | 0.0 | 0.0 | 0.0 | 0.0 | 0.0 | 0.0 | 0.6 | 0.0 | 0.0 | 0.1 |
| 5 | 0.0 | 0.0 | 0.0 | 0.0 | 0.0 | 0.0 | 0.6 | 0.0 | 0.0 | 0.1 |
| 25F or 25A | 0.0 | 2.3 | 0.0 | 0.0 | 0.0 | 0.0 | 0.0 | 0.0 | 0.0 | 0.1 |
| 35A/C | 0.0 | 0.0 | 0.0 | 0.0 | 0.0 | 0.8 | 0.0 | 0.0 | 0.0 | 0.1 |

UK = United Kingdom; in bold: serotypes of the highest prevalence in a country; NT=not typable

**Table S4:** Serotypes distribution by age category (%). Serotypes listed in order from highest to lowest in frequency. Only serotypes represented by more than 10 strains among all *S. pneumoniae* strains cultured in the study are reported. Correlation between serotypes frequency in different age groups is shown in the last column.

| **Serotypes** | **N** | **4-9 years** | **10 years or older** | **Unknown age** | **p-value** |
| --- | --- | --- | --- | --- | --- |
| 11A | 60 | 31.7 | 66.7 | 1.7 | 0.5442 |
| 23A | 58 | 34.5 | 65.5 | 0.0 | 0.8206 |
| 19A | 52 | 44.2 | 53.8 | 1.9 | 0.2606 |
| 3 | 51 | 39.2 | 60.8 | 0.0 | 0.8191 |
| 06C | 44 | 34.1 | 63.6 | 2.3 | 0.9236 |
| NT | 43 | 23.3 | 76.7 | 0.0 | 0.6331 |
| 23B | 39 | 46.2 | 51.3 | 2.6 | 0.2238 |
| 19F | 38 | 44.7 | 55.3 | 0.0 | 0.3827 |
| 23F | 37 | 56.8 | 43.2 | 0.0 | 0.0162* |
| 15B/C | 35 | 48.6 | 51.4 | 0.0 | 0.1929 |
| 35B | 34 | 35.3 | 64.7 | 0.0 | 0.6125 |
| 35F | 33 | 42.4 | 57.6 | 0.0 | 0.2522 |
| 22F | 32 | 15.6 | 84.4 | 0.0 | 0.0196* |
| 06A | 28 | 35.7 | 64.3 | 0.0 | 0.9320 |
| 10A | 28 | 42.9 | 57.1 | 0.0 | 0.6289 |
| 06B | 25 | 28.0 | 68.0 | 4.0 | 0.5723 |
| 15A | 24 | 25.0 | 70.8 | 4.2 | 0.3933 |
| 17F | 23 | 13.0 | 87.0 | 0.0 | 0.0302* |
| 24F | 21 | 57.1 | 42.9 | 0.0 | 0.0830 |
| 18C | 20 | 55.0 | 45.0 | 0.0 | 0.1393 |
| 16F | 19 | 52.6 | 47.4 | 0.0 | 0.2256 |
| 07F | 18 | 33.3 | 66.7 | 0.0 | 0.9558 |
| 09N | 17 | 29.4 | 70.6 | 0.0 | 0.7053 |
| 37 | 17 | 52.9 | 47.1 | 0.0 | 0.2893 |
| 14 | 16 | 62.5 | 37.5 | 0.0 | 0.0581 |
| 33F | 15 | 20.0 | 80.0 | 0.0 | 0.4599 |
| 21 | 13 | 38.5 | 61.5 | 0.0 | 0.8732 |
| 34 | 13 | 23.1 | 76.9 | 0.0 | 0.7831 |
| 31 | 12 | 25.0 | 75.0 | 0.0 | 0.2777 |

*) P<0.05 is significant; NT = not typable

**Table S5** Primers used in this study.

For amplification, 20 µl PCR mixtures containing HotStarTaq mastermix (Qiagen, Hilden, Germany) and 10 pmol (*wciN* and *wciP*) or 25 pmol (all others) of each primer were used for 30 cycles of amplification under the following conditions: *wciN* and *wciP*, 1 min at 95°C, 30 sec at 58°C, and 1 min at 72°C; all others, 45 sec at 95°C, 45 sec at 54°C, and 1 min at 72°C.

| **Primer** | **Sequence** | **Target** | **Used for CT's** | **Reference** |
| --- | --- | --- | --- | --- |
|  |  |  |  |  |
| wciNbeta_F | TGCACAGTACTTTTGCAGGTGT | *wciNbeta* | 06A-01, 06A-03, 06A-04, 06A-06, 06B-01, 06B-02, 06C-01 | This study |
| wciNbeta_R | CGCCCACGCAATTCGCCATC |  |  |  |
| wciP-up | ATGGTGAGAGATATTTGTCAC | *wciP* | 06A-01, 06A-03, 06A-04, 06A-06, 06B-01, 06B-02 | [7] |
| wciP-down | AGCATGATGGTATATAAGCC |  |  |  |
| 15B/C-f | TTGGAATTTTTTAATTAGTGGCTTACCTA | *wzy* | 15B-01, 15C-01, 22F-01, 23F-01 | CDC |
| 15B/C-r | CATCCGCTTATTAATTGAAGTAATCTGAACC |  |  |  |
| **35A/35C**/42-f | ATTACGACTCCTTATGTGACGCGCATA | *wzx* | 15C-01 | CDC |
| **35A/35C**/42-r | CCAATCCCAAGATATATGCAACTAGGTT |  |  |  |
| 35B-f | GATAAGTCTGTTGTGGAGACTTAAAAAGAATG | *wcrH* | 15C-01 | CDC |
| 35B-r | CTTTCCAGATAATTACAGGTATTCCTGAAGCAAG |  |  |  |
| 17F-f | TTCGTGATGATAATTCCAATGATCAAACAAGAG | *wciP* | 15C-01 | CDC |
| 17F-r | GATGTAACAAATTTGTAGCGACTAAGGTCTGC |  |  |  |
| 24F/24A/24B-f | GCTCCCTGCTATTGTAATCTTTAAAGAG | *wzy* | 24F-01 | CDC |
| 24F/24A/24B-r | GTGTCTTTTATTGACTTTATCATAGGTCGG |  |  |  |
| 20-f | GAGCAAGAGTTTTTCACCTGACAGCGAGAAG | *wciL* | 24F-01 | CDC |
| 20-r | CTAAATTCCTGTAATTTAGCTAAAACTCTTATC |  |  |  |
| 7C/7B/**40**-f | CTATCTCAGTCATCTATTGTTAAAGTTTACGACGGGA | *wcwL* | 24F-01 | CDC |
| 7C/7B/**40**-r | GAACATAGATGTTGAGACATCTTTTGTAATTTC |  |  |  |
| wzg-1_f | TGTGACAGCACCGACTGGGACT | *wzg* | 34-01 | This study |
| wzg-1_r | GTTGGTTGCGACCACGGTCACG |  |  |  |
| ST38-F | TGCCAAGACACTTGGGGAAG | *wcyV* | 25F-02 | This study |
| ST38-R | TCAGCAGGCTTCTCTATCGTCT |  |  |  |

**References**

1. De Carvalho Gomes H, Muscat M, Monnet DL, Giesecke J, Lopalco PL: **Use of seven-valent pneumococcal conjugate vaccine (PCV7) in Europe, 2001-2007**. *Euro Surveill* 2009, **14**(12).

2. Paulke-Korinek M, Kollaritsch H, Kundi M, Schmidle-Loss B, Zwazl I, Laaber B, Lakovits K, Vecsei A, Wiedermann U, Burgmann H: **Characteristics of invasive pneumococcal disease in hospitalized children in Austria**. *European journal of pediatrics* 2014, **173**(4):469-476.

3. Chlibek R, Anca I, Andre F, Cizman M, Ivaskeviciene I, Mangarov A, Meszner Z, Perenovska P, Pokorn M, Prymula R *et al*: **Adult vaccination in 11 Central European countries - calendars are not just for children**. *Vaccine* 2012, **30**(9):1529-1540.

4. **Impact of childhood pneumococcal vaccination programmes and activities for pneumococcal vaccines in the EU and EEA \EFTA countries** [venice.cineca.org/VENICE_Survey_PNC_1_2012-02-24.pdf]

5. Pebody RG, Leino T, Nohynek H, Hellenbrand W, Salmaso S, Ruutu P: **Pneumococcal vaccination policy in Europe**. *Euro Surveill* 2005, **10**(9):174-178.

6. Isaacman DJ, McIntosh ED, Reinert RR: **Burden of invasive pneumococcal disease and serotype distribution among *Streptococcus pneumoniae* isolates in young children in Europe: impact of the 7-valent pneumococcal conjugate vaccine and considerations for future conjugate vaccines**. *Int J Infect Dis* 2010, **14**(3):e197-209.

7. Mavroidi A, Godoy D, Aanensen DM, Robinson DA, Hollingshead SK, Spratt BG: **Evolutionary genetics of the capsular locus of serogroup 6 pneumococci**. *Journal of bacteriology* 2004, **186**(24):8181-8192.
